# Agonist-Biased Signaling via Matrix Metalloproteinase-9 Promotes Extracellular Matrix Remodeling

**DOI:** 10.3390/cells7090117

**Published:** 2018-08-26

**Authors:** Bessi Qorri, Regina-Veronicka Kalaydina, Aleksandra Velickovic, Yekaterina Kaplya, Alexandria Decarlo, Myron R. Szewczuk

**Affiliations:** 1 Department of Biomedical and Molecular Sciences, Queen’s University, Kingston, ON K7L 3N6, Canada; bessi.qorri@queensu.ca (B.Q.); nicka.kalaydina@queensu.ca (R.-V.K.); av36@queensu.ca (A.V.); 13yk5@queensu.ca (Y.K.); 2 Department of Biology, Biosciences Complex, Queen’s University, Kingston, ON K7L 3N6, Canada; 14ald4@queensu.ca

**Keywords:** GPCR bias agonism, MMP-crosstalk, extracellular matrix, biased signaling, functional selectivity, insulin receptor, EGFR, Toll-like receptor, GPCR

## Abstract

The extracellular matrix (ECM) is a highly dynamic noncellular structure that is crucial for maintaining tissue architecture and homeostasis. The dynamic nature of the ECM undergoes constant remodeling in response to stressors, tissue needs, and biochemical signals that are mediated primarily by matrix metalloproteinases (MMPs), which work to degrade and build up the ECM. Research on MMP-9 has demonstrated that this proteinase exists on the cell surface of many cell types in complex with G protein-coupled receptors (GPCRs), and receptor tyrosine kinases (RTKs) or Toll-like receptors (TLRs). Through a novel yet ubiquitous signaling platform, MMP-9 is found to play a crucial role not only in the direct remodeling of the ECM but also in the transactivation of associated receptors to mediate and recruit additional remodeling proteins. Here, we summarize the role of MMP-9 as it exists in a tripartite complex on the cell surface and discuss how its association with each of the TrkA receptor, Toll-like receptors, epidermal growth factor receptor, and the insulin receptor contributes to various aspects of ECM remodeling.

## 1. Introduction

The extracellular matrix (ECM) is the dynamic non-cellular structure present in all tissues and organs of the body that provides the essential physical scaffolding for cells and initiates necessary biochemical signaling required for the maintenance of tissue homeostasis [1]. Fundamentally, the ECM is composed of over 300 proteins that are collectively referred to as the core matrisome, consisting of collagen, elastin, fibronectin, proteoglycans, glycosaminoglycans, and glycoproteins [2]. However, each tissue has a unique ECM composition with specific structures that generate continuous remodeling and reciprocal signaling between them and the ECM. Collectively, these interactions have been implicated in the regulation of several vital processes, including cell survival, growth, migration, and differentiation, all of which are necessary to maintain tissue homeostasis [3].

The development and remodeling of the heterogeneous ECM rely on all cell types, including epithelial, fibroblasts, immune cells, and endothelial cells, to synthesize and secrete matrix macromolecules [3]. In addition to these cellular secretions, there are ECM-associated proteins such as growth factors, cytokines, mucins, and ECM-modifying enzymes, all of which collectively contribute to the dynamic ECM network [4]. The ECM provides a structural network for the body through binding of the ECM components to each other and other cells through receptors such as integrins, which allow for signal transduction from the ECM to cells to regulate functions that are vital to the maintenance of homeostasis [4,5]. More recently, adhesion to ECM components has been implicated in cell migration through the ECM, underlying countless critical physiological processes, including morphogenesis and wound healing, and in a more deregulated state, malignant cell invasion, and metastasis [6].

The nature of the ECM composition and structure contributing to the status of ECM remodeling has been associated with the development of several pathological conditions [3,4]. Abnormally high ECM deposition has been associated with fibrosis and cancer, whereas excessive ECM degradation has been linked to the development of osteoarthritis [7]. The intricate balance between ECM deposition and degradation in the process of ECM remodeling is mediated by enzymes degrading the matrix, such as matrix metalloproteinases (MMPs), a disintegrin and metalloproteinases (ADAMs), ADAMs with thrombospondin motifs (ADAMTSs), plasminogen activators, and heparinases during both normal and pathological conditions [4]. Of these mediators, MMPs have been studied as the primary regulators of ECM composition; however, more recently, MMPs play additional roles in controlling the interactions between cells and responses to the environment [8]. This review will focus on the important role played by MMP-9 in the process of ECM remodeling, on maintaining the architecture of various tissues, and in regulating homeostatic functions. We aim to highlight the effects of MMP-9 on ECM molecules and discuss how these effects translate to disruptions and changes in cell-matrix and cell-cell interactions in both standard and pathological conditions.

## 2. MMPs

MMPs are the primary enzymes in the cleaving components of the ECM and have been long associated with playing a central role in tissue remodeling [8]. Under normal conditions, MMP activity is negligible; however, during repair or remodeling processes such as wound healing, inflammation, or diseased tissue, MMP activity is significantly increased [9]. There are currently 23 mammalian MMPs identified that are classified into six groups: collagenases, gelatinases, stromelysins, matrilysins, membrane-type MMPs (MT-MMPs), and others by substrate specificity, sequence similarity, and domain organization [10]. All mammalian MMPs share structural similarities, characterized by a conserved catalytic metalloproteinase domain structure and an autoinhibitory prodomain that consists of the cysteine-switch motif PRCGXPD that coordinates the active-site zinc-binding motifs to prevent the functioning of the catalytic domain [10,11]. Most MMPs are secreted as zymogens and are subsequently activated in the extracellular space via proteolytic cleavage by Ser proteases or other MMPs to remove the prodomain and make the active site available for catalysis [7]. 

Two gelatinases within the MMP family, gelatinase A and B (MMP-2 and -9, respectively), contain three repeats of a fibronectin type II motif in the metalloproteinase domain, which mediate binding to collagens [10,11]. These gelatinases are capable of digesting some ECM molecules, including type IV, V, and XI collagens, laminin, and aggrecan core protein [12]. MMP-2 is known to be involved in physiological collagen turnover, whereas MMP-9 has been associated with degradation of the ECM and initiating and promoting vessel formation [13]. Due to the additional roles of MMP-9 proteolysis, particularly as it relates to regulation of tissue architecture and vascular remodeling, this review will focus on the essential roles of MMP-9 in ECM remodeling throughout the body, and the comparable mechanisms at work that rely on its activity.

MMPs are found mainly as membrane-bound or soluble secreted inactive proenzymes. The soluble forms become active in the extracellular matrix [14]. A variety of cells produce MMPs, including epithelial cells, fibroblasts, inflammatory cells, and endothelial cells. Their activities are regulated by the family of tissue inhibitors of metalloproteinase (TIMPs) [15]. Under normal and pathological conditions, MMP-1, -2, -3, -7, and -9 forms are upregulated in endothelial cells. 

The MMPs that are linked to the cell transmembrane and glycosylphosphatidylinositol (GPI)-domains are not restricted only to the plasma membrane, since the secreted forms, such as MMP-1, -2, -7, -9, -13 and -19, can also bind to the plasma membrane [16,17]. Interestingly, Fridman et al. [16] reviewed the evidence for MMP-9 docking on the cell surface, which involves a distinct array of surface proteins regulating the localization, inhibition, and internalization of the enzyme. These unique structural and functional characteristics of MMP-9 associated with cell-surface proteins provide novel conceptual challenges to its cellular functions and ECM remodeling. For example, the plasma membrane-bound MMPs can hydrolyze cell-associated substrates, activate and concentrate their activity in discrete areas, maintain their activity from inhibition, and silence the activity. To this end, MMP-9 has a high affinity for type IV collagen α2 chain [18], the intercellular adhesion molecule-1 (ICAM) [19], the beta 1-integrin in focal contacts [20], the hyaluronan receptor CD44 [21], and the scavenger receptor LRP (low-density lipoprotein receptor-related protein) [22]. From early studies, membrane-bound MMP9 represented only a very small fraction of the enzyme, while the secreted form is more biologically relevant in tumor development [23]. 

Membrane-bound MMPs can be activated or inhibited in caveolae, cholesterol-rich plasma-membrane invaginations, where MMP activators such as MMP14 can concentrate MMP2 partition in caveolae domains or rafts [24]. MMP inhibitors like GPI-anchored RECK (reversion inducing cysteine-rich protein with Kazal motifs) are also found in these rafts [25]. Thus, MMPs have multiple functional roles having substrates other than components of the ECM, and can function before invasion in the development of cancer. For example, membrane-type 1 MMP (MT1-MMP) localization at the front of migrating cells has been shown to focus its proteolytic activity in specific cellular areas [26]. MT1-MMP localizes at the front of migrating cells and degrades the extracellular matrix barrier during cancer invasion, but how the polarized distribution of MT1-MMP at the migration front is regulated was unknown until now. Mori et al. [26] have shown that MT1-MMP forms a complex with CD44H via the hemopexin-like (PEX) domain. The cytoplasmic tail of CD44H, which tethers with the actin cytoskeleton, localizes at the lamellipodia. Thus, CD44H is a linker connecting MT1-MMP to the actin cytoskeleton to direct MT1-MMP to the migration front.

Our recent studies have demonstrated another essential location of MMP-9, which exists in a complex with mammalian membrane-associated sialidase neuraminidase-1 (Neu-1) and receptor tyrosine kinases [27].

### 2.1. Matrix Metalloproteinase-9 Crosstalk with Neuraminidase-1 on Cell-Surface Receptors

We have reported that associated G-protein-coupled receptor (GPCR) signaling potentiates MMP-9-Neu-1 crosstalk, which forms a complex with various receptor tyrosine kinases (RTKs), including the nerve growth factor TrkA receptor [28], the epidermal growth factor receptor (EGFRs) [29], and the insulin receptor (IR) [30], as well as Toll-like receptors-2 and -4 (TLR-2, -4) [31]. This same receptor-signaling complex has been observed across these receptors, suggesting that MMP-9 activity is ubiquitous among these different receptors. 

Briefly, ligand binding to its specific receptor (RTK or TLR) induces GPCR-signaling processes via the Gα_i_ subunit and MMP-9 activation to induce Neu-1 activity, which collectively forms a complex with the RTK/TLR on the cell surface. Activated MMP-9 removes elastin-binding protein (EBP) to induce Neu-1 activity. Interestingly, it has been shown that during monocyte differentiation into macrophages, Neu-1 tends to relocate from lysosomes to the cell surface, but other sialidases’ (Neu-2, Neu-3, and Neu-4) expression does not change [32]. In addition to Neu-1 on the cell surface, cathepsin A, a lysosomal carboxypeptidase and EBP form a complex on the cell surface [33]. The association of Neu-1 with the multienzymatic complex containing β-galactosidase and cathepsin A [34] and EBP with the ectodomain of TLRs and RTKs is thought to be due to a unique orientation of Neu-1 on the cell surface. Neu-1 was shown to be associated tightly with a subunit of cathepsin A, with the complex influencing sialic acid levels on the cell surface of activated cells [35]. 

**Figure 1:**
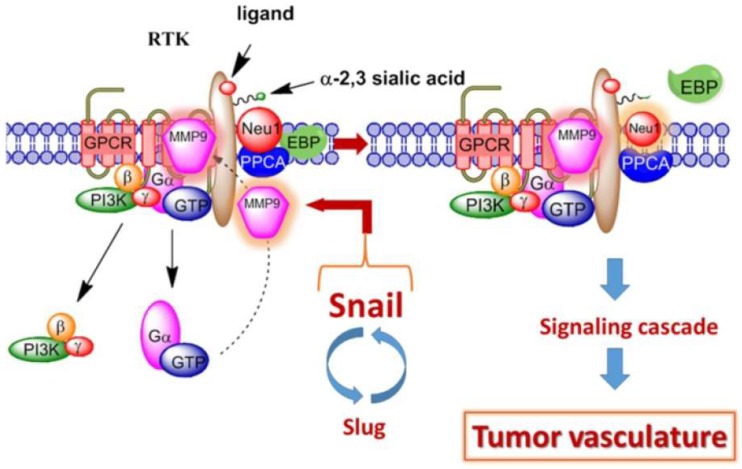
Snail and matrix metalloproteinase-9 (MMP-9) signaling axis in facilitating a neuraminidase-1 (Neu1) and MMP-9 crosstalk in regulating receptor tyrosine kinases (RTKs) to promote tumor neovascularization. Notes: For ovarian cancers, Snail and MMP-9 expressions are closely connected to similar invasive tumor processes. Snail induces MMP-9 secretion through oncogenic H-Ras (RasV12) and other multiple signaling pathways. Snail also leads to the transcriptional upregulation of MMP-9. This Snail-MMP-9 signaling axis is the connecting link in promoting the modification of growth-factor-receptor glycosylation involving the subsequent receptor-signaling platform of Neu1-MMP-9 crosstalk in complex with RTKs within their ectodomains. Activated MMP-9 removes the elastin-binding protein (EBP) within the molecular multienzymatic complex consisting of β-galactosidase/Neu1 and protective protein cathepsin A (PPCA). Activated Neu1 hydrolyzes α-2,3-sialic acid residues of RTKs to remove steric hindrance leading to receptor association and activation. Here, the stage for Snail’s role in tumor neovascularization is established. Abbreviations: GPCR, G-protein coupled receptor; Pi3K, phosphatidylinositol 3-kinase; GTP, guanine triphosphate. Citation: © 2014 Abdulkhalek et al. [37]. Licensee and published by Springer. Under the Creative Commons Attribution License, this is an Open Access article (http://creativecommons.org/licenses/by/4.0), which permits unrestricted use, distribution, and reproduction in any medium provided the original work be appropriately credited.

Activated Neu-1 hydrolyzes α-2,3-sialic acid residues on glycosylated receptors to remove steric hindrance and facilitate receptor dimerization and activation, as seen in Figure 1 [36]. This process sets the stage for downstream signaling and its involvement in multiple pathological conditions, such as tumorigenesis, inflammation, insulin resistance, and synaptic plasticity [28,29,30,31]. Figure 1 depicts for the first time the ability of transcriptional factor Snail in mediating ovarian tumor neovascularization [37]. Abdulkhalek et al. [37] provided supporting evidence to show that silencing Snail in ovarian carcinoma cells resulted in the absence of massive tumor vascularization associated with heterotopic xenografts in immunodeficient mice with a concomitant no tumor growth and metastatic burden in the lungs. Snail and MMP-9 expressions in invasive tumors like ovarian cancers are closely associated since they have similar invasive processes involving extracellular matrix remodeling [38].

Moreover, in cooperation with oncogenic RasV12 and other signaling pathways, Snail induces MMP-9 secretion through the upregulation of MMP-9 transcription [39]. Collectively, it is proposed that the Neu1-MMP-9 crosstalk may in fact be the invisible link connecting the Snail–MMP-9 signaling axis through the modification of the growth-factor-receptor glycosylation [37]. The progressive growth of a tumor to a larger size requires them to induce revascularization to ensure constant nutrient supply. Cancer cells induce ECM remodeling and local ECM reorganization to acquire additional tumor space, thereby promoting tumor growth. 

Indeed, extracellular matrix remodeling and cellular changes in adhesion molecules are necessary for a cancer cell to become motile. Rearrangement of the actin cytoskeleton promotes cell motility and plasticity together with downregulation of adhesion molecules, which facilitate the binding to the ECM. Following ECM binding, integrins can activate MMP-9 synthesis and regulate its expression [40]. 

**Figure 2:**
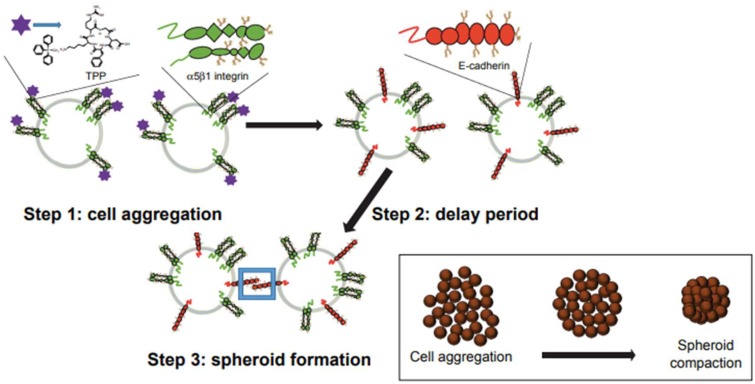
Formation steps of MCTS by the cyclo-RGDfK(TPP) peptide-based biochemical method. Step 1: formation of loose cell aggregates via α5β1 integrin—cyclo-RGDfK(TPP) peptide binding; step 2: a delay period for E-cadherin expression and accumulation; step 3: formation of compact MCTS through E-cadherin—E-cadherin interactions. Abbreviations: MCTS, multicellular tumor spheroid; cyclo-RGDfK(TPP), cyclic Arg-Gly-Asp-d-Phe-Lys peptide modified with 4-carboxybutyl-triphenylphosphonium bromide. Citation: © 2017 Haq et al. [45]. Licensed and published by Dove Medical Press Limited. Noncommercial reproduction of the work is permitted without any further permission from Dove Medical Press Limited, provided the work be cited appropriately.

Recognition of surface integrins followed by the extracellular matrix, collectively termed integrin-guided proteolysis, is an essential mechanism in cell invasion and cancer metastasis [41]. The α3β1 integrin is coexpressed with MMP-9 in epithelial-cell carcinomas as well as in epithelial wound healing, with α3β1 signaling required for the sustained production of MMP-9. The sustained MMP-9 expression is responsible for the invasive nature of tumor cells [42]. The bidirectional signaling of integrins in mediating cell-to-ECM interactions involves an arginine-glycine-aspartic acid (RGD) binding motif that has led to our understanding of tumor growth using the ECM remodeling. The RGD motif is characteristic of fibronectin, a significant component of the ECM [43]. To this end, we have engineered a peptide consisting of cyclic Arg-Gly-Asp-d-Phe-Lys (cycloRGDfK) conjugated with triphenylphosphonium cation (TPP), referred to as cyclo-RGDfK(TPP), which we have previously reported to form 3D multicellular spheroids [44]. Briefly, cyclo-RGDfK(TPP) peptide interacts with α5β1 integrins on the cell surface. This interaction stimulates expression of E-cadherin, an adhesion molecule which has been shown to facilitate the formation of compact, tight spheroids (Figure 2). The report also proposed that these cyclo-RGDfK(TPP) peptides mimic the natural ECM protein’s ability to induce cell aggregation via α5β1 integrin. Interestingly, the relative levels of specific sialoglycan structures on the cell surface correlated significantly with the ability of the cancer cells to form avascular multicellular tumor spheroids as well with xenograft tumors.

### 2.2. Integrins and Matrix Metalloproteinase-9

Integrins are transmembrane receptors that facilitate cell–ECM adhesion. Activation of integrins by their respective ligands induces signal cascades that mediate the reorganization of the intracellular cytoskeleton during the cell cycle, providing movement of new receptors to the cell membrane. Integrins are heterodimeric transmembrane adhesion receptors composed of α and β subunits that mediate bidirectional cell-to-cell and cell-to-ECM interactions [46]. Interestingly, integrins can interact with MMPs to induce multiple signaling pathways that modulate cell proliferation, differentiation, and migration [47]. For example, MMP-9 has been found to play a crucial role in long-term potentiation (LTP) of the nervous system, synaptic plasticity, and maintenance of neuronal dendritic spines [48,49,50,51]. Multiple mechanisms have been proposed to explain MMP-9-induced ECM remodeling within the nervous system with a particular focus on integrin receptors [49,52]. 

When the classical integrin-binding motif arginine-glycine-aspartic acid (RGD) binds to the β1 subunit of integrin receptors, increased surface diffusion of the *N*-methyl-d-aspartate receptor (also known as the NMDA receptor or NMDAR) occurs [52]. The NMDA receptor controls synaptic plasticity and memory function. It is a specific type of ionotropic glutamate receptor because NMDA selectively binds to it, and not to any other glutamate receptors. NDMA receptors also coexpress with integrins, implicating them in synaptic plasticity [53]. RGD-containing ligands binding to integrins require MMP-9 activity to expose the RGD motif, also known as the integrin-activating epitope [51]. It is noteworthy that the tropomyosin receptor kinase A (TrkA) receptor and α9β1 integrins are coexpressed on the same cells [54]. Thus, MMP-9 may be involved in regulating neuronal growth and maintenance through the TrkA- and β1 integrin-signaling pathways. 

### 2.3. Matrix Metalloproteinase-9 and the TrkA Receptor

The TrkA receptor is a high-affinity neurotrophin RTK associated with nerve growth factor (NGF) binding. It maintains neuronal function, including development, survival, maturation, proliferation and synaptic plasticity of neuronal cells [55,56,57,58,59,60]. The TrkA receptor is homologous to other Trk receptors, consisting of a transmembrane domain, an intracellular tyrosine kinase domain, and a five-subunit extracellular domain. The extracellular domain includes a leucine-rich region (LRR) that is flanked by two cysteine-rich subdomains with two immunoglobulins (Ig)-like subdomains, Ig-C1 and Ig-C2 [61]. NGF binding to TrkA at the Ig-C2 subdomain results in receptor homodimerization and autophosphorylation, which subsequently activate the receptor [57]. The mechanism(s) of this neuronal TrkA homodimerization and autophosphorylation signaling by NGF was unknown until now. We reported that NGF binding to its receptor induces membrane-associated sialidase activity that hydrolyzes α-2-3-sialyl residues of TrkA receptors [62]. This desialylation process is the initial step for receptor dimerization, internalization, and subsequent activation of TrkA-expressing neuronal cells as well as primary cortical neurons [62]. The subsequent development of neurite outgrowth in neuronal cells and their survival responses against cell death caused by oxidative stress, hypoxia-induced neurite retraction, and serum/glucose deprivation, is regulated by this modification of receptor glycosylation process [63]. Collectively, a prerequisite desialylation of Trk receptors by the membrane sialidase enables the removal of steric hindrance to receptor association. Jayanth et al. [28] have identified Neu1 as the membrane sialidase involved in the mechanism initiated by NGF binding to TrkA. The NGF binding to its receptor potentiates an unprecedented GPCR signaling via membrane Gαi subunit proteins and MMP-9 activation to induce the activation of Neu1 sialidase in live primary neurons and TrkA- and TrkB-expressing cell lines, as depicted in Figure 1. Central to this activation process is that the Neu1/MMP-9 complex is bound to TrkA on the cell surface of naïve primary neurons and Trk-expressing cells. These findings support the concept of MMP-9 and Neu1 crosstalk playing a crucial role in regulating NGF-induced TrkA activation. This receptor-desialylation process mediated by Neu1 activation is depicted in Figure 1. Here, these receptors include TrkA [28,62], IR [30,64,65], insulin growth factor receptor-1 (IGF-R1) [65], TLRs [27,31,66,67], EGFR [29], and others [68,69]. Pshezhetsky and Hinek [69] reported a new dimension for cellular signaling and molecular targeting, which involves the desialylation of cell-surface receptors. 

### 2.4. G protein-Coupled Receptors Biased Agonism to Activate TrkA

It is well known that the association of GPCR and RTK signaling including Trk and insulin receptors upon ligand binding is eloquently reviewed by Pyne and colleagues [70,71,72], Abdulkhalek et al. [36], and Haxho et al. [73]. Onfroy et al. [74] proposed a mechanism dictating biased agonism involving G protein stoichiometry through distinct partitioning of receptor-G protein integration. Here, the expression levels of Gα proteins influence the biased profiling of β-agonists and antagonists by affecting different membrane distribution of receptor-G protein populations, in that they determine both their activity and efficacy. The level of Gα expression in the naïve state influences the partitioning of not only Gα but also the coexpressed receptor in different membrane domains [74]. Indeed, GPCRs can select more than one active state that is called ‘biased agonism’, ‘functional selectivity’, or ‘ligand-directed signaling’ [75,76]. Similarly, an array of allosteric ligands can have different degrees of modulation where they facilitate ‘biased modulation’ and can vary dramatically in a probe- and pathway-specific manner [75,77,78]. This biased modulation is not due to differences in orthosteric ligand efficacy or stimulus-response coupling. 

The phenomenon of GPCR-biased agonism has led to an increased interest in the potential mechanism(s) of transactivation of the TrkA receptor. GPCR pleiotropy has resulted in the concept of biased agonism offering the potential to exploit cell functioning to achieve desired effects through cell signaling while avoiding those that are linked to adverse effects [79]. For example, adenosine and pituitary adenylate cyclase-activating polypeptides (PACAP) have been identified as GPCR ligands that elicit comparable downstream neurotrophic effects in the absence of neurotrophins [80,81]. Adenosine binding to A2A GPCR has been shown to result in the activation of the phosphatidylinositol 3-kinase (PI3K)/Akt pathway, involved in regulating neuronal survival [80,82]. This signaling activity may be due to adenosine-mediated activation of the TrkA receptor, which may have clinical implications in the treatment of neurodegenerative diseases, such as Alzheimer’s disease. Recently, it has been demonstrated in animal models that Alzheimer’s disease may be associated with a shortage of endogenous NGF supply [4]. Based on this association, various therapies have focused on the use of exogenous NGF to treat and mitigate the symptoms of Alzheimer’s. However, there are obstacles associated with the use of neurotrophic molecules, which stem from difficulties in their delivery and pharmacokinetics [83]. The idea of allosteric modulators has been proposed to confer agonist-biased GPCR signaling and selectively modulate specific signaling pathways while having little to no effect on other parallel pathways [84]. Therefore, the exploitation of agonist adenosine through GPCR-biased agonism could instead be used to create a novel therapy that will not only aid in regulating and slowing the progression of Alzheimer’s and potentially other neurodegenerative diseases but will also promote ECM remodeling of injured tissue.

### 2.5. TrkA, MMP-9, and Angiogenesis 

In addition to acting as a neurotrophic factor in the nervous system, NGF also plays a role in the induction of angiogenesis [85,86,87]. Angiogenesis is the formation of new vascular networks from pre-existing ones throughout embryonal development and in several physiological and pathological conditions, such as in wound healing, inflammation, and cancer and metastasis [88]. NGF was shown to promote tissue healing in limb ischemia, the results of which demonstrated a neural-drive mechanism for angiogenesis [89]. NGF-mediated angiogenesis is due to expression of the TrkA receptor on the surface of endothelial cells and vascular smooth muscle cells [90,91]. NGF was found to potentiate reparative angiogenesis in diabetic wounds and stimulating epithelial-cell proliferation [92]. NGF binding to TrkA results in activation of intracellular signaling cascades including the protein kinase ERK pathway, PI3K/Akt pathway, and phospholipase C pathway that promote cell-cycle progression [92,93]. MMP-9 plays an essential role in angiogenesis in which ECM remodeling permits migration of endothelial cells and smooth muscle cells throughout the tissue, and promotes the release of sequestered growth factors, including angiogenic growth factors [88,94]. TrkA activation by NGF binding in smooth muscle cells induces MMP-9 expression, which is required to degrade the ECM surrounding the smooth muscle to promote migration [94]. Vessel remodeling requires ECM alterations that are a result of MMP-9 activity. The RGD site expressed in fibronectin and other ECM proteins that can bind to integrins has been shown to impact epithelial cell adhesion, migration, proliferation, survival, and cell-to-cell interactions that occur during angiogenesis [95].

Recently, Su et al. [96] have reviewed neurotrophins and their receptors involved in regulating tissue formation and healing in skeletal tissues. Here, neurotrophin NT-3 ligand, which specifically binds the TrkC receptor, can be an osteogenic and angiogenic factor. NT3 is known to enhance the expression of the essential osteogenic factor, BMP-2, and the major angiogenic factor, vascular endothelial growth factor (VEGF), to promote bone formation, vascularization, and healing of the injury site [97]. Saran et al. [98] reviewed the connection of vascularization during bone healing and remodeling, and the insight into the current therapeutic strategies adapted to promote angiogenesis. Bone repair and remodeling together are essential for the activation as well as the interaction between angiogenic and osteogenic signaling pathways. Interestingly, angiogenesis precedes the onset of osteogenesis.

### 2.6. MMP-9 and EGFR Signaling

The EGFR is an RTK receptor that is implicated in pathways that regulate cell survival, proliferation, and differentiation of mammalian cells [99]. The EGFR belongs to the ErbB family of RTKs and is comprised of an extracellular domain-binding ligand, a hydrophobic transmembrane domain, and a cytoplasmic domain that contains tyrosine kinase activity [100]. Once epidermal growth factor (EGF) ligand binds to the extracellular domain of the EGFR, this induces the formation of homo- or heterodimers, which subsequently activate cytoplasmic tyrosine kinase activity [100,101]. Several ligand precursors for the EGFR are produced as membrane-bound proteins that require cleavage to be released [102,103,104]. MMPs facilitate the release of several EGFR ligands from the cell surface, implicating MMPs with the invasiveness of tumor growth [105,106]. In this way, MMP overcomes ECM sequestering of growth factors, and can locally release factors including EGF, fibroblast growth factor (FGF), and VEGF [4]. 

### 2.7. Biased Agonism of G-Protein Coupled Receptors to Activate EGFRs

Ligand-dependent or ligand-independent mechanisms can mediate transactivation of EGFR. Ligand-dependent transactivation requires the cleavage and release of EGF ligands by paracrine or endocrine mechanisms, whereas ligand-independent activation relies on GPCR kinases [107]. Interestingly, activation of EGFR upregulates MMP-9, which, in turn, degrades E-cadherin, a crucial facilitator of cell–cell adhesion and differentiation. This crosstalk has been implicated in many cancer types, particularly in the development of metastasis [108,109]. Transactivation of EGFR following ligand binding to a GPCR has been shown to involve pro-HB-EGF and MMP activity that is rapidly induced following the ligand binding to the GPCR [104]. Gilmour et al. [29] have identified a novel molecular EGFR-signaling paradigm. Here, Neu1 and MMP-9 are found already in complex with naïve EGFRs, and are rapidly activated by EGF stimulation of the receptor. Neu1 specifically hydrolyzes α-2,3-sialyl residues that are distant from ligand binding; this process enables removal of receptor steric hindrance to association, activation, and subsequent signaling pathways. Furthermore, the neuromedin B GPCR is found to be associated with EGF-induced Neu1 activity in live 3T3–hEGFR cell line. This novel Neu1 and MMP-9 crosstalk, together with GPCR neuromedin B, is an essential signaling platform for EGF-induced receptor activation and cellular signaling. Indeed, Moody et al. [110] have also reported that the EGF-receptor transactivation is regulated by neuromedin B GPCR, a mechanism dependent on Src as well as MMP activation. In support of this EGFR-signaling paradigm, Lillehoj et al. [111] have provided evidence to show that Neu1 associates with EGFR as well with the cell-surface-associated mucin-1 (MUC1) in respiratory-airway epithelial cells. EGF stimulation regulates this Neu1–EGFR association, and when tested in vivo, it played a role in airway epithelial repair, wound healing, tumorigenesis, and metastatic potential [111]. Elevated EGFR levels are associated with reduced disease outcomes in several cancer types, including non-small-cell lung carcinoma (NSCLC), ovarian cancer, and head and neck cancer [112]. MMP-9 activity is essential for the release of EGFR ligands in preovulatory ovarian follicles stimulated with pituitary peptide hormone-luteinizing hormone (LH), and in gonadotropin-releasing hormone-stimulated (GnRH) gonadotropic cells [113].

### 2.8. MMP-9 and IR Signaling

The IR is a transmembrane tyrosine kinase receptor that is activated by insulin and insulin growth factors I and II [114]. The IR is a heterotetrameric protein consisting of two extracellular α subunits joined by disulphide bonds to two transmembranes β subunits. These subunits are generated from a large precursor molecule by proteolytic cleavage [115]. Similarly to other ligand-binding induced activations discussed, insulin binding to its receptor results in a conformational change that induces activation of kinase activity in the IR-β subunits [116]. The resulting downstream effects of IR activation involve glucose homeostasis and broader pleiotropic actions due to IR expression on a wide range of cells, including liver, skeletal muscle, fat, and brain cells [117,118]. Insulin resistance as a result of decreased sensitivity to the ligand and decreased ligand availability presents as a significant problem in many pathological conditions [118]. The precise mechanism(s) involved in insulin resistance is not well understood [119]. However, insulin resistance may be due to increased plasma-free fatty acid levels [120], elastin-derived peptides [121], subclinical chronic inflammation [119], oxidative and nitrative stress, altered gene expression, and mitochondrial dysfunction [119,120].

**Figure 3:**
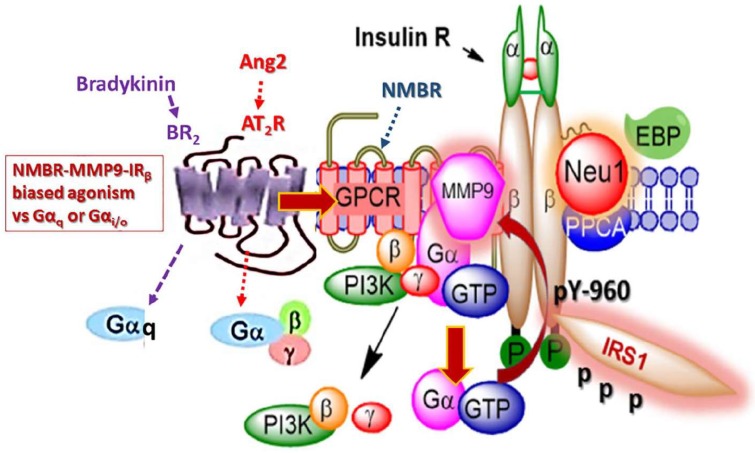
Mechanism of GPCR bias agonism involves insulin receptor activation. Bradykinin and angiotensin II form a complex with the neuromedin B receptor (NMBR), IRβ, and Neu-1. Bradykinin and angiotensin II preferentially lead to insulin-receptor signaling by first forming a complex with NMBR. This heterodimerization leads Gα, β, and γ to activate MMP-9. Upon activation, MMP-9 removes EBP, which in turn activates Neu-1. Crosstalk between these activated components leads to the phosphorylation and subsequent activation of insulin receptor substrate 1 (IRS1), initiating the phosphoinositide 3-kinase-protein kinase B (PI3K–AKT) pathway, in addition to others, without insulin. Citation: Taken from Haxho et al. [127] with permission, Cellular Signaling, Volume 43, Issue 6, March 2018, Pages 71–84. 0898-6568/© 2017 Published by Elsevier Inc., Open access under CC BY-NC-ND license. The article is an Open Access article that permits unrestricted noncommercial use, provided the original work be appropriately cited.

Recently, Lukong et al. [34] proposed that insulin binding to its receptor rapidly facilitates the interaction of Neu1 sialidase with the insulin receptor, which then hydrolyzes sialic acid residues of IR and, consequently, induces receptor activation. They showed that Neu1-deficient mice exposed to a high-fat diet developed hyperglycemia and insulin resistance twice as fast as the control cohort. Based on these results, endogenous Neu1 sialidase activity plays an important role in the regulation of the insulin receptor. Blaise et al. [121] provided additional evidence to support that Neu1 interacts with IRβ and desialylates the receptor. Alghamdi et al. [30] provided additional evidence for a novel GPCR signaling platform regulating the IRβ subunits. Here, the findings in their report showed data to support a novel Neu1 and MMP-9 crosstalk in alliance with neuromedin B GPCR tethered to IRβ subunit on the cell surface, all of which are essential for insulin-induced IR activation and cellular signaling. It is noteworthy that insulin can mediate increases in MMP-9 via IR activation [122], in support of the molecular-signaling platform of Neu1-MMP-9 crosstalk in alliance with neuromedin B GPCR in regulating insulin-induced receptors as depicted in Figure 3. GPCR and IR have also been shown for β-adrenergic receptors tethered to IR in adipocytes [123,124,125,126]. Here, insulin binding IR stimulates the phosphorylation of the β-adrenergic receptor on Tyr-350, the process of which facilitates IR tethering to β-adrenergic receptor via growth factor receptor-bound protein 2 (Grb-2). This molecular IR/β-adrenergic receptor/Grb-2 tripartite complex is essential for β-adrenergic agonist amplification of insulin-dependent activation of p42/p44 MAPK.

### 2.9. Biased Agonism of G-Protein-Coupled Receptors to Activate the Insulin Receptor 

More recently, Haxho et al. [127] provided strong supporting evidence for the novel concept of biased GPCR agonist-induced IRβ transactivation signaling axis (Figure 3). GPCRs are ubiquitous throughout the body and participate in numerous physiological processes, making them important targets for therapy [128]. To that end, Haxho et al. [127] found that bradykinin (BR2) and angiotensin II receptor type I (AT2R) exist in a multimeric receptor complex with neuromedin B, IRβ, and Neu1 in naïve and stimulated IR-expressing HTC cells. This novel molecular-signaling platform regulates the interaction and signaling mechanism(s) between these players on the cell surface, uncovering the missing link for a biased GPCR agonist-induced IRβ transactivation-signaling axis, mediated by Neu1 sialidase and the modification of insulin receptor glycosylation. The biased GPCR-signaling platform potentiates Neu1 and MMP-9 crosstalk on the cell surface. This signaling platform was deemed essential for the activation of the IRβ tyrosine kinases. Indeed, biased GPCR agonism may, therefore, contribute to the mechanism of ECM remodeling occurring due to upregulation of MMP-9 activity. For instance, MMP activity has been reported to increase during chronic hyperglycemia resulting in excess degradation of tissue matrix [129]. A prominent clinical manifestation of MMP-9-induced ECM remodeling is diabetic retinopathy in type 2 diabetes mellitus (T2DM). This phenomenon of GPCR bias agonism has been observed with the use of various agonists towards the activation of several receptors and presents the potential for novel therapeutics [130,131]. For example, diabetic retinopathy (DR) is a complication of T2DM resulting from persistent hyperglycemia that leads to chronic subclinical inflammation, with long-term effects on the vascular dysfunction of the retinal microvasculature [132]. The ensuing visual loss associated with diabetic retinopathy is attributed to the increased permeability of retinal vessels or due to the proliferation of new retinal vessels [133]. Here, MMP-9 would weaken the blood–retinal barrier, thereby breaking down endothelial tight junction protein cadherin and occluding in the early stages of diabetic retinopathy [134,135]. As such, MMP-9 is considered to be a critical mediator of retinal ischemia-induced angiogenesis and nonperfusion-mediated tissue injury [129]. Preclinical studies have shown that high levels of glucose can result in transcriptional overexpression of MMP-9 [136,137,138]. Elevations of MMP-9 have been recorded in diabetic individuals, with both T2DM and diabetic retinopathy [139], and correlated with increased severity of T2DM [140]. Recently, Jayashree reported that MMP-9 was shown to be significantly increased in patients with T2DM with diabetic retinopathy compared to individuals with T2DM without retinopathy [129]. The proinflammatory state that exists in patients with diabetic retinopathy was suggested to cause overexpression of MMP-9 [129]. However, in light of recent advances in our understanding of GPCR-biased agonism, perhaps another explanation could be that increased levels of GPCR agonists are contributing to the activation of MMP-9, which could partially account for its role in diabetic retinopathy.

### 2.10. IR and Cardiac Extracellular Matrix Remodeling

Concerning GPCR-biased agonism, angiotensin I and II agonists previously mentioned have been directly implicated in ECM remodeling [141]. Here, angiotensin I and II are known to be involved in cardiac remodeling by acting through the AT1 and AT2 receptors. Left-ventricular remodeling has been associated with the activated renin–angiotensin–aldosterone system (RAAS), while the inhibition of RAAS has been shown to mitigate ventricular remodeling in a failing heart [142]. Changes in the extracellular collagen matrix of the myocardium are crucial to the remodeling process following acute myocardial infarction [143]. At the cellular level, ECM remodeling has been suggested to be observed due to increased myocyte hypertrophy, fibroblast hyperplasia, and increased collagen deposition [144]. Ducharme et al. [145] showed that targeted deletion of the MMP-9 gene attenuated left-ventricular enlargement after experimental myocardial infarction (MI) in mice with decreased collagen content. More recently, Zheng et al. [146] have shown that renal sympathetic denervation (RSD) may improve postmyocardial infraction through upregulation of TIMP-1 in rats. A higher concentration of MMP-9 and decreased TIMP-1 protein expression in cardiac tissue following MI has been postulated to result in proteolytic imbalance [146]. A possible explanation for RSD improving cardiac remodeling following MI could be attributed to the development of a feed-forward loop whereby the tumor growth factor β1 (TGF-β1) increases MMP-9 and MMP-2; however, both MMP-9 and MMP2 can also cleave latent TGF-β1 and release its active form, which activates the transcription of TIMPs [147]. Given that patients suffering from insulin resistance are at an increased risk of cardiovascular pathology [148], ECM remodeling following MI is of particular importance in the context of diabetes. The open link between GPCR-biased agonism and the IR may further explain the development of severe cardiovascular pathology in insulin-resistant patients, as reviewed by Liauchonak et al. [149]. Furthermore, cardiac ECM remodeling in the context of insulin-resistance may be occurring through biased GPCR agonist-IR crosstalk in part due to the activity of MMP-9. 

### 2.11. Matrix Metalloproteinase-9 and TLRs

TLRs are pattern-recognition receptors (PRRs) that can recognize a broad range of molecular patterns including exogenous pathogen-associated molecular patterns (PAMPs) and endogenous damage-associated molecular patterns (DAMPs) [150]. TLRs are highly conserved type I transmembrane glycoproteins that consist of an extracellular domain, containing leucine-rich repeat (LRR) motifs, and an intracellular Toll/interleukin-1 (IL-1) receptor (TIR) domain [150]. Cell surface TLRs function to recognize extracellular microbes (TLR-1, -2, -4, -5, and -6) or intracellular TLRs localized within the endosomal compartment of the cell that are known to recognize nucleic acids (TLR-3, -7, -8, and -9) [151]. Collectively, TLRs play critical roles in the immune response, particularly in inflammation and tissue damage. 

**Figure 4:**
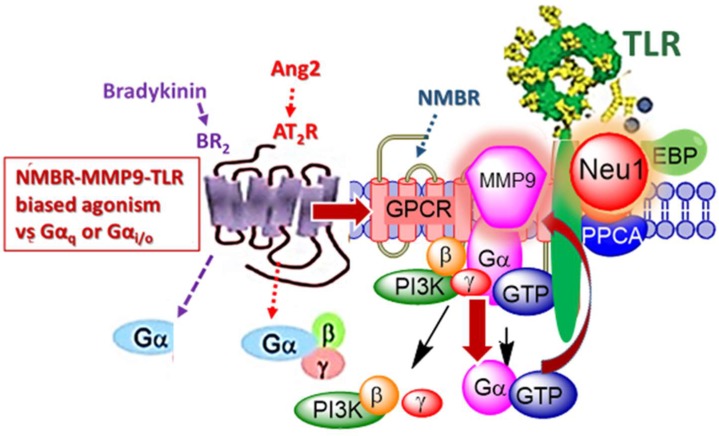
Bradykinin (BR2) and Angiotensin II receptor type I (AT2R) exist in a multimeric receptor complex with NMBR, IRβ, and Neu1 in naïve (unstimulated) and stimulated RAW-blue macrophage cells. Here, a molecular link regulating the interaction and signaling mechanism(s) between these molecules on the cell surface uncover a biased GPCR agonist-induced cell surface and intracellular Toll-like receptor (TLR) transactivation-signaling axis, mediated by Neu1 sialidase and the glycosylation modification of TLRs. The biased GPCR-signaling platform here potentiates Neu1 and MMP-9 crosstalk on the cell surface that is essential for the transactivation of TLRs and subsequent cellular signaling. Notes: TLR ligand, as well as GPCR agonists, can potentiate biased NMBR-TLR signaling and subsequently induce MMP-9 activation and Neu1 sialidase activity. Activated MMP-9 is proposed here to remove the EBP as part of the molecular multienzymatic complex that contains β-galactosidase/Neu1 and PPCA. Activated Neu1 then hydrolyzes α-2,3 sialyl residues of TLR at the ectodomain to remove steric hindrance to facilitate TLR association and subsequent recruitment of MyD88 and downstream signaling. Citation: Adapted from Abdulkhalek et al. [66].

Ligand binding to TLRs induces receptor oligomerization and, in turn, dimerization of the intracellular domain, which subsequently triggers the activation of downstream-signaling cascades [150]. The TIR domain is required for complex formation with four activating adapter molecules, myeloid differentiating factor-88 (MyD88), MyD88 adapter-like (Mal), TIR domain-containing adapter-inducing IFN-β (TRIF), and TRIF-related adapter molecule (TRAM) [151]. The binding adapters induce downstream activation of transcription factors, including NF-κB and type I interferons (IFN) [151]. MMP-9 has been shown to contribute to TLR activation, by further instigating the stimulation of an inflammatory response, with TLR activation being recognized as playing a role in chronic pain initiated by the immune signaling [152]. Indeed, studies have provided evidence to show that CpG oligodeoxynucleotide (ODN) induces transcriptional TNFα and TNFR-II expressions, which are involved in the expression of MMP9 in the supernatants derived from murine macrophage cell line by a TLR9 and a serine/threonine-specific protein kinase B (Akt)-mediated mechanism [153,154]. This ODN-induced MMP9 expression fits well within our novel Neu-1 and MMP9 crosstalk regulating TLR receptors depicted in Figure 4 and reported by Abdulkalek et al. [27]. That report describes the key players involved in the activation of nucleic acid sensing intracellular TLR-7 and TLR-9 receptors against imiquimod and CpG oligodeoxynucleotide (ODN), respectively [27].

### 2.12. Biased G-Protein-Coupled Receptors Agonism to Activate TLRs

Conformational changes induced by ligand binding of bombesin-related neuromedin B receptor to TLR-7 and -9 allows for the initiation of GPCR signaling through membrane-bound MMP-9 activation by the Gαi subunits [27]. GPCR-mediated MMP-9 activation, in turn, induces Neu-1 sialidase, creating a three-part complex with TLR-7 and -9, which triggers TLR dimerization and recruitment of MyD88 adapter molecule [27]. A similar crosstalk is seen in the TLR-4 receptor when binding endotoxin lipopolysaccharide (LPS) [31]. Once again, ligand-induced TLR conformational changes trigger GPCR signaling through MMP-9 and Gαi subunits and, in turn, induce Neu-1 sialidase activity [31]. The interplay of these molecules and receptors are essential to creating a unique molecular signaling platform that is required for ligand-induced TLR activation as seen in Figure 4 [31]. These studies further demonstrate the biased agonism or functional selectivity phenomenon, as GPCR appear to favor activation of the unique downstream pathway by different ligands [155]. 

### 2.13. TLR Induced MMP-9 Activity in Chronic Inflammation

TLR4 is known to promote a proinflammatory response due to its primary ligand binding, LPS [156]. LPS promotes the release of vasoactive inflammatory mediators from vascular smooth muscle cells, thought to contribute to chronic inflammation. MMP-9 is known to be involved in smooth muscle cell migration, and LPS binding to TLR4 was found to upregulate MMP-9 expression in human aortic smooth muscle cells [157]. 

Angiotensin II (Ang II) has been shown to play a proinflammatory role in the development of atherosclerosis. Ang II was shown to upregulate TLR-4 expression in cells and upregulate MMP-9 activity. This finding suggests that Ang II stimulates inflammation in vascular smooth muscle cells through regulation of inflammatory factors in an Ang-II-dependent manner [158]. The proinflammatory effects of Ang II have been thoroughly studied, with Ang II promoting immune system activation by upregulating TLR-4. However, a mechanism remains to be elucidated [159,160]. Thus, these proinflammatory effects of Ang II may be the result of bias of the angiotensin II receptor (AT2R) to activate TLR-4 to stimulate tumor necrosis factor-α (TNF-α) preferentially and induce NF-κB activity. Similar results have been obtained with bradykinin, whereby bradykinin upregulates TLR-4 expression and promotes an inflammatory response [161].

Cellular fibronectin is produced in response to tissue injury and contains an extra domain A (EDA). EDA-containing fibronectin produces cellular responses similar to those induced by bacterial LPS. EDA was found to activate TLR-4 and persisted in the absence of LPS antagonists. These findings suggest a mechanism by which EDA-containing fibronectin promotes an inflammatory response [162]. To this end, in the context of airway smooth muscle (ASM) contractility/relaxation, GPCR receptors alone, or together with receptor tyrosine kinases, can contribute to the functionality of ASM involving cellular proliferation, growth, and subsequent secretion of growth factors and inflammatory mediators. These processes will thus influence airway remodeling and the local inflammatory milieu as eloquently reviewed by Prakash [163]. Surrounding the cells in the airway, there is a network of collagenous and noncollagenous ECM protein structures, whereby the density and the composition of these ECM structures can influence the cellular functions such as proliferation, migration, differentiation, and survival. The ECM alone can regulate the formation and release of growth factors and MMPs where they can modify several extracellular proteins involved in ECM remodeling. For example, alterations in ECM asthmatic-airway remodeling can involve enhanced deposition of collagens I, III, and V, fibronectin, tenascin, hyaluronan, versican, and perlecan with a concomitant decrease in other proteins such as collagen IV and elastin [164]. In addition, this altered ECM asthmatic airway could facilitate inflammatory mediators produced by surrounding cells including growth factors. Here, ASM not only responds to inflammatory mediators but is also a source of a wide variety of pro- and anti-inflammatory factors. This ASM immunomodulatory function and property of ASM can be induced by inflammation, infection and microbial products [163]. 

## 3. Conclusions

Matrix metalloproteinase-9 plays a crucial role in the remodeling of the extracellular matrix. With the novel discovery of receptor transactivation or GPCR agonist-bias signaling, MMP-9 has been shown to play a more prominent role in ECM remodeling. Through activation of associated GPCRs, such as the angiotensin AT1R and bradykinin receptors, MMP-9 is activated to induce activation of RTKs such as the TrkA receptor, EGFR, and IR as well as Toll-like receptors in the absence of their respective ligands. These discovered roles of MMP-9 provide countless options for novel therapeutic targets in the treatment of pathological conditions including cancer, atherosclerosis, diabetes, inflammation, and wound healing. It is noteworthy that the work highlighted here is by no means all-encompassing of the substantial body of the recent literature.

## Abbreviations

ADAM	a disintegrin and metalloproteinase
ADAMTS	ADAMs with thrombospondin motifs
Akt	protein kinase B
Ang II	angiotensin II
ASM	airway smooth muscle
AT2R	angiotensin II receptor type I
BR2	bradykinin
cycloRGDfK	cyclic Arg-Gly-Asp-D-Phe-Lys
DAMP	damage-associated molecular pattern
DR	diabetic retinopathy
EBP	elastin binding protein
ECM	extracellular matrix
EDA	extra domain A
EGF	epidermal growth factor
EGFR	epidermal growth factor receptor
FGF	fibroblast growth factor
GnRH	gonadotropin-releasing hormone
GPCR	G protein-coupled receptor
GPI	glycosylphosphatidylinositol
Grb-2	growth factor receptor-bound protein 2
H-RAS	RasV12
ICAM	intercellular adhesion molecule-1
IFN	type I interferon
Ig	immunoglobulin
IGF-R1	insulin growth factor receptor-1
IL-1	interleukin-1
IR	insulin receptor
IRS1	insulin receptor substrate 1
IRβ	insulin receptor β subunit
LH	leuteinizing hormone
LPS	lipopolysaccharide
LRP	low density lipoprotein receptor-related protein
LRR	leucine-rich region
LTP	long-term potentiation
Mal	MyD88 adaptor-like
MI	myocardia infarction
MMP	matrix metalloproteinase
MMP-9	matrix metalloproteinase-9
MT-MMP	membrane-type MMP
MT1-MMP	membrane-type 1 MMP
MUC1	mucin-1
MyD88	myeloid differentiating factor-88
Neu-1	neuraminidase-1
NGF	nerve growth factor
NMBR	neuromedin B receptor
NSCLC	non-small cell lung cancer
ODN	oligodeoxynucleotide
PACAP	pituitary adenylate cyclase-activating polypeptide
PAMP	pathogen-associated molecular pattern
PEX	hemopexin-like
PI3K	phophatidylinositol 3-kinase
PI3K-AKT	phosphoinositide 3-kinase-protein kinase B
PPCA	protective protein cathepsin A
PRR	pattern-recognition receptor
RAAS	renin-angiotensin-aldosterone system
RECK	reversion inducing cysteine-rich protein with Kazal motifs
RGD	arginine-glycine-aspartic acid
RSD	renal sympathetic denervation
RTK	receptor tyrosine kinase
T2DM	type 2 diabetes mellitus
TGF-β1	tumor growth factor β1
TIMP	tissue inhibitors of metalloproteinase
TIR	Toll/interleukin-1 receptor
TLR	Toll-like receptor
TLR-2	Toll-like receptor-2
TLR-4	Toll-like receptor-4
TNF-α	tumor necrosis factor-α
TPP	triphenylphosphonium cation
TRAM	TRIF-related adaptor molecule
TRIF	TIR domain-containing adaptor-inducing IFN-β
TrkA	tropomyosin receptor kinase A
VEGF	vascular endothelial growth factor
